# High Density Surface Electromyography Activity of the Lumbar Erector Spinae Muscles and Comfort/Discomfort Assessment in Piano Players: Comparison of Two Chairs

**DOI:** 10.3389/fphys.2021.743730

**Published:** 2021-12-01

**Authors:** Alejandra Aranceta-Garza, Alessandro Russo, Samuel D’Emanuele, Francesca Serafino, Roberto Merletti

**Affiliations:** ^1^Biomedical Engineering, School of Science and Engineering, University of Dundee, Dundee, United Kingdom; ^2^Laboratory for Engineering of the Neuromuscular System (LISiN), Department of Electronics and Telecommunications, Politecnico di Torino, Turin, Italy; ^3^Department of Neurosciences, Biomedicine and Movement Sciences, University of Verona, Verona, Italy; ^4^School of Exercise and Sport Sciences, University of Turin, Turin, Italy; ^5^Montecatone Rehabilitation Institute S.p.A., Imola, Italy

**Keywords:** surface electromyography, high-density sEMG, back muscles, piano players, musculoskeletal questionnaire

## Abstract

**Introduction:** At a professional level, pianists have a high prevalence of playing-related musculoskeletal disorders. This exploratory crossover study was carried out to assess and compare quantitatively [using high density surface electromyography (HDsEMG)], and qualitatively (using musculoskeletal questionnaires) the activity of the lumbar erector spinae muscles (ESM) and the comfort/discomfort in 16 pianists sitting on a standard piano stool (SS) and on an alternative chair (A-chair) with lumbar support and a trunk-thigh angle between 105° and 135°.

**Materials and Methods:** The subjects played for 55 min and HDsEMG was recorded for 20 s every 5 min. For the quantitative assessment of the muscle activity, the spatial mean of the root mean square (RMS_*ROA*_) and the centroid of the region of activity (ROA) of the ESM were compared between the two chairs. For the qualitative assessment, musculoskeletal questionnaire-based scales were used: General Comfort Rating (GCR); Helander and Zhang’s comfort (HZc) and discomfort (HZd); and Body Part Discomfort (BPD).

**Results:** When using the A-chair, 14 out of 16 pianists (87.5%) showed a significantly lower RMS_*ROA*_ on the left and right side (*p* < 0.05). The mixed effects model revealed that both chairs (*F* = 28.21, *p* < 0.001) and sides (*F* = 204.01, *p* < 0.001) contributed to the mean RMS_*ROA*_ variation by subject (*Z* = 2.64, *p* = 0.004). GCR comfort indicated that participants found the A-Chair to be “quite comfortable,” and the SS to be “uncomfortable.” GCR discomfort indicated that the SS caused more numbness than the A-Chair (*p* = 0.05) and indicated the A-Chair to cause more feeling of cramps (*p* = 0.034). No difference was found on HZc (*p* = 0.091) or HZd (*p* = 0.31) between chairs. Female participants (*n* = 9) reported greater comfort when using the A-Chair than the SS (*F* = 7.09, *p* = 0.01) with respect to males. No differences between chairs were indicated by the BPD assessment.

**Conclusion:** It is concluded that using a chair with lumbar support, such as the A-chair, will provide greater comfort, less exertion of the ESM and less discomfort than the standard piano stool.

## Introduction

Pianists are a small professional category with a high prevalence of playing-related musculoskeletal disorders (PRMD; Zaza, 1998; Ciurana Moñino et al., 2017). They are subjected to daily intensive use of their upper extremities whilst engaging the muscles of trunk and back, including the erector spinae muscles (ESM). This engagement makes pianists vulnerable at developing PRMD and associated symptoms such as pain and numbness (Kok et al., 2013), which negatively impact their performance (Chan et al., 2014).

Playing the piano at a professional level implies efforts due to the intensity of the practice which requires great precision, speed, accuracy and associated psychological stress due to the highly competitive environment (Quarrier, 1993; Rozmaryn, 1993; Ciurana Moñino et al., 2017). In particular, pianists have a PRMD prevalence that ranges from 26 to 96% whilst 25 to 43% experience it before even starting their music degree (Spahn et al., 2004; Amaral Corrêa et al., 2018). Musicians deal with PRMD by adapting to their everyday pain and discomfort, as they consider it not to be strong enough to affect their current abilities to play (Zaza, 1998).

Several studies discuss PRMD risk factors, however, the results of the interventions to reduce these factors have been quantified in only a few cases (Grieco et al., 1989; De Smet et al., 1998; Bragge et al., 2006; Bruno et al., 2008; Honarmand et al., 2018) using single electrode pairs. A systematic review performed by Bragge et al. (2006) identified risk factors associated with PRMD. Other studies indicate that properly shaped chairs with lumbar support are preferable to the standard piano stools (SS) and may reduce PRMD in pianists (Grieco et al., 1989; Honarmand et al., 2018). This has been further explored on other types of musicians (Foxman and Burgel, 2006; Cattarello et al., 2018; Russo et al., 2019) concluding that chairs with appropriate lumbar support and a trunk-thigh angle of 115° ± 10°, demonstrated to preserve the physiological spine lordosis angle corresponding to a high-perceived comfort (Keegan, 1953; Bendix and Biering-Sørensen, 1983), and might reduce the activity of back muscles; however, quantitative and qualitative assessments and comparisons are still lacking. The need for further research associating these elements is evidenced by recent studies and systematic reviews (Kenny and Ackermann, 2015; Berque et al., 2016).

Previous studies investigated the ESM of sitting workers and their pain mechanism using individual surface electromyography (sEMG) electrode pairs (van Dieen et al., 2001; Mork and Westgaard, 2009) whilst other authors used electrode grids of up to 128 contacts (Falla et al., 2014; Ringheim et al., 2014; Abboud et al., 2015). In a previous preliminary study (Cattarello et al., 2018), biomechanical and short term (5 min) high density surface electromyography (HDsEMG) measurements were used to compare violinists and pianists sitting on a series of different chairs (Varier Move and Varier HAG with and without lumbar support) and on a standard orchestra chair. A further study based on these findings (Russo et al., 2019) concluded that, in violinists, the A-chair (same as the one used in this study) was associated to a lower amplitude of HDsEMG of the ESM, without changes of the spatial and temporal patterns of muscle activity.

Comfort and discomfort can be assessed through subjective evaluations using scale-ranked questionnaires. These scales allow for a qualitative description of what is considered comfortable and uncomfortable for each individual (Helander, 2003). These evaluations are based on the participant’s prior knowledge/experience, current well-being, opinions, and biases. Well-known questionnaire-based methodologies to explore comfort and discomfort are the General Comfort Rating scale (GCR), the Helander and Zhang’s comfort (HZc) and discomfort (HZd), and the Body Part Discomfort scale (BPD) which assesses the comfort/discomfort by body part.

The purpose of this exploratory research was to assess and compare quantitative measurements and qualitative evaluations obtained from 16 pianists continuously playing for 55 min while sitting on a SS in day 1 and on the A-chair in day 2. This exploratory crossover study is the first using a grid of 128 sEMG electrodes on the ESM on each side of the spine of pianists as well as using qualitative outcome measures and the interaction/correlation between them.

## Materials and Methods

### Subjects and Protocol

Sixteen pianists (nine females, seven males; one professor and 15 students), all self-reported as right dominant, participated in the study. The participants had at least 5 years of professional piano playing and played, on average, (15.7 ± 7.63) hours per week. None of them reported low back pain symptoms. All musicians provided informed consent prior to the tests. All the procedures used in this study were performed in accordance with the Helsinki Declaration of 1975, as revised in 2000 and 2008, and approved by the Italian National Health Service. Table 1 shows the demographic data of the sampled population. Sex, years of experience, and weekly practice are reported for completeness; differences related to these factors have not been investigated in this study.

**TABLE 1:** Demographic and anthropometric data of the 16 pianists and their musical experience.

Subject	Sex	Age (years)	Body mass (kg)	Height (m)	BMI (kg/m^2^)	SAT thickness (mm)	Musical career (years)	Weekly practice (h/wk)
1	M	15	50	1.76	16.14	4.6	5	11
2	M	61*	78	1.74	25.76	7.0	50*	7
3	M	16	70	1.93	18.79	7.1	11	7
4	F	15	60	1.70	20.76	11.1	8	14
5	F	26	74	1.68	26.22	7.5	20	14
6	M	37	70	1.87	20.02	12.6	15	14
7	F	17	60	1.60	23.44	10.4	8	12
8	F	20	55	1.70	19.03	10.8	12	14
9	M	19	50	1.70	17.30	6.0	8	7
10	M	27	69	1.75	22.53	11.0	15	34
11	F	17	52	1.65	19.10	7.2	13	11
12	F	15	48	1.58	19.23	7.5	5	11
13	F	21	40	1.60	17.30	8.3	15	18
14	F	25	55	1.65	22.53	8.4	16	18
15	F	24	57	1.69	19.10	9.2	20	21
16	M	28	58	1.68	20.55	9.3	17	32
Mean	9F, 7M	24	59	1.71	20.49	8.63	13	15
St. dev.		11	10	0.09	2.82	2.08	5	8

*Subcutaneous adipose tissue (SAT) thickness was measured at the T11-L3 levels of the ESM. SAT Thickness is the mean of 6 measurements on the right side and 6 measurements on the left side of each subject. Body mass index (BMI) is defined as: BMI = Weight (kg)/Height^2^ (m^2^). All subjects indicated right-hand dominance.*

*Indicates an outlier value not included in the calculation of (mean and st. dev.) of age, musical career, and weekly practice.

**FIGURE 1:**
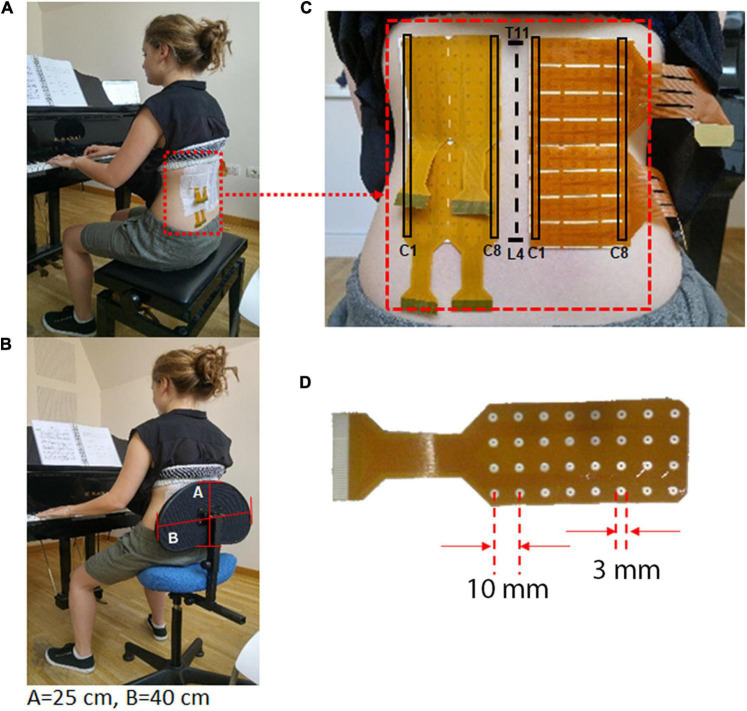
**(A)** Pianist playing on the SS (standard piano stool) keeping the trunk erect with a trunk-thigh angle of 90°; **(B)** Pianist playing on the A-Chair keeping the trunk erect, with a trunk-thigh angle between 105° and 135°. The pianist back is always in contact with the lumbar support which has a dimension of A = 25 cm by B = 40 cm; **(C,D)** An example of electrode grid positioned on the lumbar portion of the right and left ESM (between spinal processes T11 and L4). The grids have an inter-electrode distance = 10 mm and electrode diameter Ø = 3 mm. The first (C1) and last (C8) columns of the electrode grids are indicated.

Two chairs were tested on two different days, at least one week apart: the standard piano stool (SS) and the alternative chair (A-Chair). The A-Chair was a modified version of the Varier Move model with an added adjustable lumbar support providing a trunk-thigh angle of 115° ± 10°. The height of the SS and the lumbar support of the A-Chair were adjusted by the researchers to guarantee optimal position and comfort, for each pianist at the start of each session (Figures 1A,B). The trunk-thigh angle was measured with a hand goniometer by our expert physiotherapist (FS) and our expert kinesiologist (SD’E).

The pianists played continuously during 1 h a set of standard exercises. Firstly, the E major scale, moto retto, four octaves, three velocities followed by the E major scale, moto contrario, two octaves, average velocity, two modalities (low intensity increasing in the ascending phase and high intensity decreasing in the ascending phase). These exercises were repeated in an alternating order for the 55 min.

Every 5 min the pianists switched to a standard music piece, well-known by pianists of all ages and experience (first tempo of Mozart sonata N16, K 545 in C major). This piece was played for 20 s before switching back to the standard exercises. As such, a total of twelve 20-s recordings of HDsEMG were acquired. At the end of each session, the pianists completed questionnaires exploring their comfort/discomfort.

The response of each subject to the two chairs was assessed in two ways:

(1) Quantitative assessment of HDsEMG of the ESM during each of the twelve 20 s recordings; and
(2) Qualitative assessment using different musculoskeletal questionnaires exploring comfort and discomfort in different ways.

### Electrode Placement, High Density Surface Electromyography Recording and Processing

Prior to positioning of the HDsEMG grids, the skin was cleaned with abrasive paste (NuPrep, Weaver and Company, United States) and cleaned with a wet cloth to avoid paste bridges between electrodes. The grids were then placed as indicated in Figure 1C, on each side of the spine at the lumbar level on the ESM using T11 and L4 spinous processes as anatomical landmarks to ensure consistency across participants and testing sessions. The expert physiotherapist in manual therapy (FS) and our expert kinesiologist (SD’E), manually located these body landmarks using multiple methods to improve accuracy (Robinson et al., 2009; Snider et al., 2011). The electrode grids were then placed with the medial column 1 cm laterally to the spinous process, on the thoraco-lumbar muscle region identified through palpation during a lumbar extension movement. Each grid had a total of 128 electrodes (16 rows × 8 columns per array) on each side of the back. The electrodes had a diameter of 3 mm and an inter-electrode distance of 10 mm (Figure 1D), as suggested in recommendations for best practices (Merletti and Muceli, 2019).

Monopolar HDsEMG signals were collected and differentiated along the column direction, approximately in the ESM fiber direction, with respect to a reference electrode placed on the knee. An amplifier of up to 400 channels (Quattrocento, OT Bioelettronica, Turin, Italy) was used, (second order analog band-pass filter with bandwidth of 10–500 Hz, CMRR = 95 dB, input impedance >90 MΩ over the entire bandwidth, 16-bit A/D conversion, sampling frequency = 2048 Hz, gain = 500, input referred noise level <1 μV_*RMS*_ and input resolution of 0.5 μV). The differential signals were further digitally filtered with a fourth order Butterworth bidirectional (non-causal) band-pass filter with high pass cut off at 20 Hz and low pass cutoff at 400 Hz.

Background noise was estimated by performing a separate test with the same protocol on five additional subjects lying prone and relaxed on a bed for 1 h. HDsEMG signals were recorded with the same setup and procedure used for the pianists. The 12 spatial means of the background noise maps ranged from 1.90 μV_*RMS*_ to 3.30 μV_*RMS*_ with a mean of 2.61 μV_*RMS*_ and a standard deviation of 0.46 μV_*RMS*_. Hence, the background noise level was conservatively taken as 5.0 μV_*RMS*_ (about twice the average value and 1.5 times the maximum value).

When the spatial average of the RMS values of the channels (pixels) of the entire map was higher than the noise level (5 μV_*RMS*_), a Region of Activity (ROA) was defined using the Active Contour Segmentation algorithm (Caselles et al., 1997) available on Matlab v10 (The MathWorks Inc., Natick, MA, United States). This RMS spatial average over the ROA was defined as RMS_*ROA*_. Otherwise the RMS_*ROA*_ was defined over the entire map.

**FIGURE 2:**
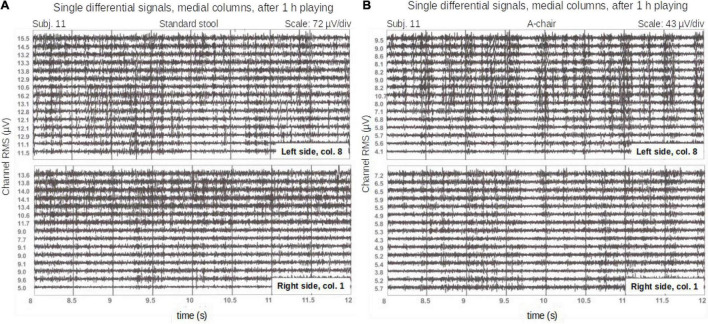
Single differential signals from a pianist (column 8 of the left grid and column 1 of the right grid) on a time window of 4 s on the standard stool **(A)** and the A-Chair **(B)**. These signals were recorded after 55 min of continuous playing. The RMS values of each channel over the entire length of the signal (20 s), are reported next to each trace. Note the different scales on the signal plots.

The RMS_*ROA*_ values were small (range from 3.39 μV_*RMS*_ to 20.39 μV_*RMS*_, with peak-to-peak sEMG values of the channels in a range of 50–200 μV) and power line interference was evident. Power line interference was removed using the spectral interpolation technique (Mewett et al., 2004). This technique was applied by (1) computing the Fourier transform of each 20-s signal, (2) removing harmonics in 10 frequency windows (centered on the first 10 harmonics of the power line) between 48 and 52 Hz, 96 and 104 Hz, 144 and 156 Hz and so on up to the 10th harmonic, (3) replacing the removed harmonics with new harmonics obtained by interpolation between previous and subsequent harmonics, (4) applying the inverse Fourier transform to re-obtain the “cleaned” signal in the time domain. ECG interference, visible in the monopolar signals, was substantially absent in the differential signals. Examples of differential signals (after spectral interpolation), obtained from column 8 of the left grid and column 1 of the right grid are provided in Figure 2.

**FIGURE 3:**
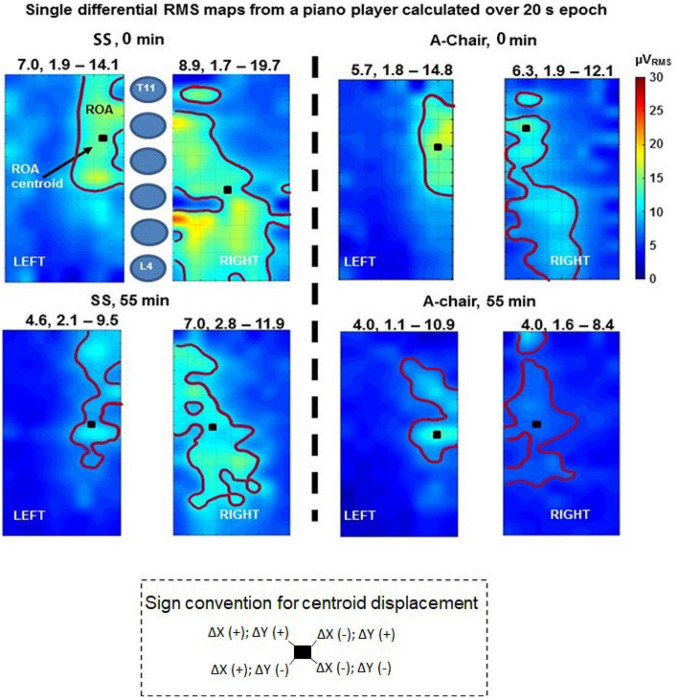
Single differential RMS maps relative to subject 15 for chair SS (left side panels) and A-chair (right side panels) at the beginning (0 min) and the end (55 min) of the test. Maps were computed on the entire 20 s length of the signals. An image interpolation by a factor of 15 was applied (1 pixel = 0.6 mm). The region of activity (ROA) is identified by means of map segmentation (Caselles et al., 1997). Above each map the mean, minimum and maximum values of surface electromyography (sEMG) over the entire map are reported (μV_*RMS*_). The centroid of each ROA, the color scale (0–30 μV_*RMS*_) and a schematic representation of the vertebrae (T11-L4) are indicated. The sign convention for centroid displacements is described at the bottom, a shift if X_*CM*_ to the left is in the lateral direction (away from the spine), for the left map, whereas it is in the medial direction (toward the spine) for the right side map.

Topographical images of the longitudinal differential HDsEMG RMS values (estimated on a 20 s epoch) provided by each grid were obtained and the ROA was computed using Caselles et al. (1997) method for segmentation. Examples of these images are provided in Figure 3 where the spatial mean of the RMS values within the contoured ROA is the RMS_*ROA*_.

### Quantitative and Qualitative Statistical Analyses

EMG measurements were performed on the RMS maps over the twelve trials (20 s every 5 min during 55 min) and used to investigate and compare chair-specific spatial and time patterns of the images. The quantitative outcome measures used were:

(1) *Mean RMS values*: As indicated in section “Electrode Placement, HDsEMG Recording and Processing,” these values were computed as the spatial means over the ROAs and defined, on the differential RMS maps, as RMS_*ROA*_. The Mann–Whitney test was used to investigate differences between RMS_*ROA*_ associated to sides and chairs, and Mixed Effects Models were used to explore the RMS_*ROA*_ variations for each subject using the two chairs as well as for the entire group of 16 subjects. Mixed effects models allowed to better evaluate the RMS_*ROA*_ variations than conventional ANOVA with the correlated error and have previously been used on values obtained from sEMG signals (Boccia et al., 2019). The choice of this method is important when using repeated measures, as the RMS_*ROA*_ values are correlated within a subject across the 12 tests. This approach treats the effects of the fixed factors (chairs and sides) separately from the random effects (pianist and time). Interaction effects were also investigated to understand the population’s trend over time regardless of statistical significance. Finally, RMS_*ROA*_ change over time [defined as: 100 × (RMS_*ROA*_ at the end of the trials–RMS_*ROA*_ at the start of the trials)/(RMS_*ROA*_ at the start of the trials)] were quantified and compared by participant using Wilcoxon signed-rank test (with chair as a factor). When statistically significant differences were found, *post hoc* Dunn’s Multiple Comparison tests were carried out.
  Finally, an interaction effect analysis was performed between RMS_*ROA*_, chairs, trials and pianists to understand how much each chair affected the activity of the muscles, and how this relation was influenced by the other variables.
(2) *Centroid of the ROA compared by chair, side and time (X_*CM*_, Y_*CM*_ coordinates)*: The coordinates X_*CM*_ and Y_*CM*_ of the center of mass, or centroid, of a distribution of mass over a surface (in our case the distribution of mass is replaced by the distribution of the RMS over the ROA region) were defined as:

$$X_{C\,M}=\frac{\sum_{i=1}^{M}\sum_{J=1}^{N}X_{i,j}\cdot I\,\left(i,j\right)}{\sum_{i=1}^{M}\sum_{j=1}^{N}I\,\left(i,j\right)},Y_{C\,M}=\frac{\sum_{i=1}^{M}\sum_{J=1}^{N}Y_{i,j}\cdot I\,\left(i,j\right)}{\sum_{i=1}^{M}\sum_{j=1}^{N}I\,\left(i,j\right)}$$

where *x* and *y* are the discrete coordinates of pixel (i,j) in mm defined with respect to the origin located in the top left corner of the map. I (i,j) is the intensity of pixel (i,j); and M and N are the total number of rows and columns, respectively. This computation only includes the pixels within the ROA; the pixels outside of the ROA were not computed in the summation.

The difference in displacement of the centroid of the A-Chair with respect to the SS, for the left (L) and right (R) sides was calculated as:


ΔL(X,Y)=[(XC⁢M⁢o⁢f⁢(A-C⁢h⁢a⁢i⁢r)-S⁢Sat 55min

-XC⁢M⁢o⁢f⁢(A-C⁢h⁢a⁢i⁢r)-S⁢Sat 0min),

(YC⁢M⁢o⁢f⁢(A-C⁢h⁢a⁢i⁢r)-S⁢Sat 55min

-YC⁢M⁢o⁢f⁢(A-C⁢h⁢a⁢i⁢r)-S⁢Sat 0min)],

and


ΔR(X,Y)=[(XC⁢M⁢o⁢f⁢(A-C⁢h⁢a⁢i⁢r)-S⁢Sat 55min

-XC⁢M⁢o⁢f⁢(A-C⁢h⁢a⁢i⁢r)-S⁢Sat 0min),

(YC⁢M⁢o⁢f⁢(A-C⁢h⁢a⁢i⁢r)-S⁢Sat 55min

-YC⁢M⁢o⁢f⁢(A-C⁢h⁢a⁢i⁢r)-S⁢Sat 0min)]

The distribution of the X_*CM*_, Y_*CM*_ was Gaussian according to the Kolmogorov–Smirnov test. An additional ANOVA multivariate analysis was applied to explore the effects of chair, side, and time on the centroid to (a) identify significant changes in the location of the ROA centroid in time and by side of the ESM, and (b) test if the coordinates of the centroid were significantly affected by the chairs on each side of the back. An image interpolation by a factor of 15 was applied (1 pixel = 0.6 mm).

The qualitative assessment was performed using different musculoskeletal questionnaire-based evaluations that were compared between the two chairs. The statistical assessment was performed treating the evaluation outcomes as continuous variables (Beam and Wieand, 1991; Nevill et al., 2002; Harpe, 2015). These outcomes were:

(1) *General Comfort Rating (GCR)*: This index explores the current musician status by a 1–10 scoring scale where 1: relaxed, 2: comfortable, 3: quite comfortable, 4: not very comfortable, 5: uncomfortable, 6: restless, 7: tight, 8: stiff, 9: numb, 10: in pain. The values reported were Gaussian distributed according to Kolmogorov–Smirnov test treated as continuous variables and comparisons were carried out through one-way ANOVA with chair as factor. When statistically significant differences were found, *post hoc* Tukey tests were carried out.
(2) *Helander and Zhang’s measures of comfort (HZc) and discomfort (HZd)*: These indicators have seven and nine statements, respectively. Each statement was ranked from 1 to 10, where 1 was “do not agree” and 10 was “totally agree”. This non-linear assessment of comfort/discomfort was analyzed by individual statements between chairs as a percentage of comfort and discomfort (Zhang et al., 1996). The 1–10 values reported on these scales were treated as continuous variables and were Gaussian-distributed according to the Kolmogorov–Smirnov test. The measures were compared between chairs (across pianists) using paired-sample *t*-tests.
(3) *Body Part Discomfort (BPD) rating*: This index explores if there is pain or discomfort on 10 different body parts individually, ranking them from 0 to 10, where 0 was “no pain or discomfort” and 10 was “extreme pain or agony.”

Body part discomfort comparisons were performed between chairs (across pianists) using General Linear Mixed Models (GLMM). Dunn’s *post hoc* was applied to allow multiple comparison adjustments.

The relationship between quantitative and qualitative measurements was explored using Spearman’s correlation. This was done by correlating the RMS_*ROA*_ changes in time between chairs (across subjects), with each qualitative measure obtained (GCR, HZc, HZd, and BPD). This representation is only indicative since the 10 grades of each scale cannot be assumed to represent equally spaced values of comfort/discomfort.

All the statistical analyses were carried out using Minitab v19 (Minitab LLC, PA, United States).

### Measurement of Subcutaneous Adipose Tissue

Subcutaneous adipose tissue thickness is known to reduce the sEMG RMS values (Kuiken et al., 2003). SAT thicknesses were measured by three operators, and checked for differences between three measurement sites (the ESM region was divided into three sub-regions at T11, L1, L3 levels) and between left and right side, using an ultrasound scanner (Echo Blaster 128, Telemed, Lithuania). Since the Kolmogorov–Smirnov test confirmed the Gaussian distribution of the SAT values, the two-way ANOVA was used to assess significant differences between sites (across participants). The correlation between SAT and RMS amplitude was then investigated using Pearson’s correlation coefficient.

### Statistical Analyses

The research question addressed in this work aims at assessing changes of sEMG RMS values and of subjective perception of comfort and discomfort induced by using the A-Chair compared to using the SS in 16 subjects. Twelve 20 s measurements were performed during 55 min of play, 5 min apart, on each subject for both chairs. Statistical tests concerning sEMG RMS values were the same as adopted in previous work (Russo et al., 2019) and based on the Wilcoxon signed-rank test. Dunn’s *post hoc* tests were used to allow for multiple comparisons adjustments.

The qualitative assessment was carried out through questionnaire-based Likert-type evaluation which contained more than five categories. As such, an ordinal approximation of a continuous variable was used, and the evaluation of the outcomes of these scales was treated as a continuous variable (Beam and Wieand, 1991; Nevill et al., 2002; Harpe, 2015).

## Results

### Raw Signals Quality and Amplitude

The spatial mean of RMS computed over the ROA (if RMS value >5 μV), or over the entire map (if RMS value <5 μV) ranged from 3.39 to 20.39 μV_*RMS*_. An example of the signal quality is shown on a 4 s recording window (out of the 20 s) in Figure 2. Examples of RMS maps are shown in Figure 3.

In seven subjects (44%) out of 16, the signals from most electrode pairs, presented visually evident burst-like activity patterns as observed previously (Cattarello et al., 2018; Russo et al., 2019) on the same muscles. These bursts were observed to last 100–390 ms and repeating about 1.5–2.5 times per second. They are barely visible in Figure 2B. These bursts were not related to either side or chair and were not investigated further as this was not the aim of this work.

### Quantitative Changes in High Density Surface Electromyography Signals

Typical topographical maps of the RMS values computed over the right and left grids at the start (recording 1, at 0 min) and end (recording 12, at 55 min) obtained from both chairs are shown in Figure 3. The mean RMS_*ROA*_ was different between chairs. The effect of the chair on the RMS_*ROA*_ of each map was quantified by the mean (over 12 measurements) percentage change of the RMS_*ROA*_ for each subject sitting on the A-Chair with respect to the SS, defined as –100 [RMS_*ROA*_(A-Chair)–RMS_*ROA*_ (SS)]/RMS_*ROA*_ (SS). Wilcoxon ranked sign tests with Dunn’s *post hoc* were used to compare the median differences for each pianist (*N* = 16), computed across trials (*N* = 12) between chairs (*N* = 2), for each side. These comparisons (Table 2) showed that 14 (87.5%) of the 16 subjects had statistically greater values of the RMS_*ROA*_ on the SS than on the A-Chair on both left and right sides. As mentioned in section “Discussion,” these results are in agreement with Wilcoxon signed-rank tests carried out on violinists (Russo et al., 2019).

**TABLE 2:** Mean percentage change of the RMS_*ROA*_ for each subject sitting on the SS and A-Chair.

Subject	Mean RMS_*ROA*_ % change due to chair type on left side (mean ± st. dev.) *N* = 12	Mean RMS_*ROA*_ % change due to chair type on right side (mean ± st. dev.) *N* = 12
1	−10.48 ± 12.05*	18.17 ± 15.08*
2	51.08 ± 4.60**	24.88 ± 7.05**
3	33.43 ± 14.89**	41.43 ± 10.04**
4	25.63 ± 12.09**	21.80 ± 13.18**
5	34.18 ± 4.22**	45.07 ± 5.51**
6	−13.53 ± 13.19*	0.66 ± 10.85
7	49.88 ± 3.75**	59.46 ± 1.78**
8	21.70 ± 8.21**	22.26 ± 8.21**
9	59.81 ± 2.27*	53.26 ± 2.82**
10	29.59 ± 5.96**	18.36 ± 5.10**
11	22.74 ± 15.47*	52.62 ± 3.00**
12	38.17 ± 12.43**	59.38 ± 5.79**
13	60.04 ± 7.67**	56.11 ± 6.15**
14	20.65 ± 11.98**	33.26 ± 6.78**
15	44.10 ± 6.70**	62.24 ± 4.46**
16	12.73 ± 7.24**	3.49 ± 8.65

**Total**	29.69 ± 23.01**	35.47 ± 21.46**

*The means of the % changes were tested using Wilcoxon signed-rank test with post hoc Dunn’s tests where appropriate, for being significantly positive or negative. All but two decrements were positive on the left side. For each subject the mean and st. dev. of 100⋅(RMS_SSi_ – RMS_Ai_)/RMS_SSi_ is computed for (1 < i < 12), where i is the index of the measurements performed every 5 min, over a 20 s epoch, for 55 min. The total RMS % change is the pooled mean across the 16 pianists.*

*Denotes RMS_ROA_ % difference between chairs as being significantly different from 0 with p < 0.05, and ** with p < 0.01.

The A-Chair was generally associated to lower RMS_*ROA*_ amplitude throughout the trials when compared to the SS. This lower amplitude reflects less involvement of the ESM whilst using the A-Chair. This observation was further confirmed when changes of the RMS_*ROA*_ were assessed for each pianist by chair and side of the ESM (Table 2). The mean percent difference of the RMS_*ROA*_ across all pianists was (35.47 ± 21.46) % on the right side and (29.69 ± 23.01) % on the left side. This difference between sides was significantly different from zero (Wilcoxon signed-rank test with *post hoc* Dunn’s test).

The mixed effects model was applied to assess the RMS_*ROA*_ change in time individually and across the sample population to compute the differences taking fixed and random factors into consideration (fixed factors: chairs and sides; random factors: pianist and time). The statistical tests revealed that both chairs (mixed effects model, *F* = 423.18, *p* < 0.001) and sides contributed to the RMS_*ROA*_ variation with the right side presenting greater RMS amplitude than the left side (*F* = 21.09, *p* < 0.001). When these factors were assessed for each subject, the individual RMS_*ROA*_ differences were confirmed to be influenced by the chairs and side (*Z* = 2.69, *p* = 0.004). A global decreasing trend in time of RMS_*ROA*_ was observed on both chairs, with a mean difference over time between chairs of 2.80 μV with (RMS_*ROA*_ of SS) > (RMS_*ROA*_ of A-Chair). The model goodness-of-fit explained 96.97% (R^2^) of the RMS_*ROA*_ with a st. dev. of 0.70. This highlights that the changes on the RMS_*ROA*_ were directly influenced by the chairs and sides of the ESM.

#### Centroid of the Region of Activity

The averaged displacement difference (A-Chair – SS) of the CM over 55 min [mean, (range)] for the right side was ΔR (X,Y): [−1.74, (−17.30, 24.00), −1.69, (−6.67, 9.33)] mm; and for the left side ΔL (X,Y): [−1.33, (−22.67, 15.33), −1.33, (−18.33, 13.33)] mm. In Figure 3, the ROA centroid is shown for each of the RMS maps shown as well as the sign convention adopted to describe its displacement over 55 min. The ANOVA multivariate analysis (fixed factors: chair and side; random effect: pianists and time) was applied to identify if there were significant changes in the location of the centroid over time (at the 12 different timepoints, one for each of the 12 trials), and to test if its location shifted significantly depending on the chair used. No significant change attributable to chairs in the displacement or location of the CM on either side of the spine was found.

### Qualitative Changes in the Musculoskeletal Questionnaire-Based Assessment

All the results from the questionnaires were Gaussian-distributed as confirmed by the Kolmogorov–Smirnov test unless otherwise explicitly mentioned.

#### General Comfort Rating

The A-Chair was considered most comfortable (mode = 3: “quite comfortable”) when compared to SS, which was found to be mostly uncomfortable (mode = 5: “uncomfortable”) (one-way ANOVA, *p* < 0.001).

#### Helander and Zhang’s Comfort Statements

**FIGURE 4:**
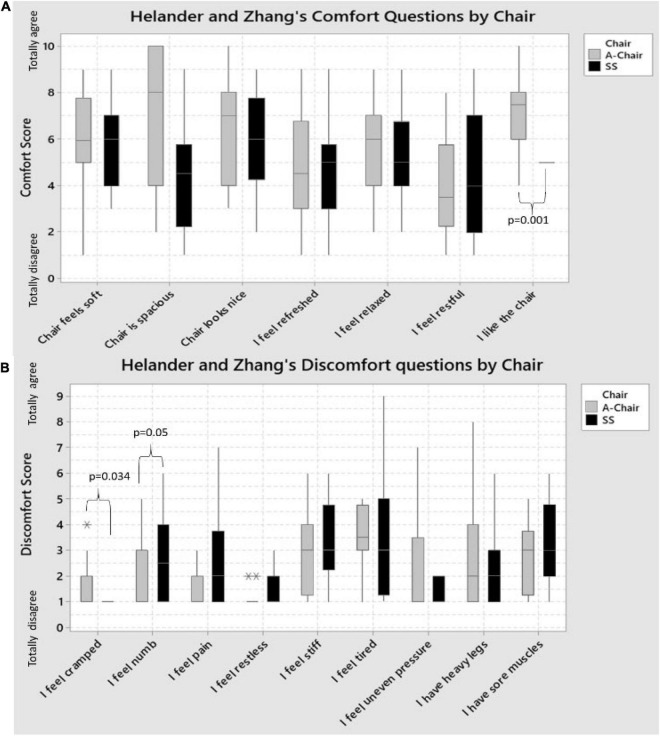
Boxplots showing the Helander and Zhang’s statements of: **(A)** Comfort; and **(B)** Discomfort for the SS and A-Chair on the 16 pianist. For most Comfort questions (statements), the A-Chair had a better or equal score than the SS. The only exception was the restfulness of the chair: more pianists felt the SS was better than the A-Chair. The only statistically significant difference between chairs was “I like the chair,” with A-Chair being significantly more liked, than the SS. The sitting on the A-Chair was perceived as significantly “cramped” when compared to the SS (*p* = 0.034), whilst the SS was perceived to provide greater numbness feeling (*p* = 0.05) than the A-Chair. For the rest of the discomfort questions, no statistically significant difference was found, however, in five of the remaining seven statements, the pianists agreed the SS had a greater discomfort than with the A-Chair. Statistical outliers are indicated with * and **.

Paired-sample *t*-tests were run on the seven statements for the 16 pianists to determine whether there were statistically significant mean differences in comfort when using the A-Chair versus the SS (Figure 4A). From the seven statements, only one “I like the chair” presented a statistically significant difference between chairs with pianists “mostly agreeing” to “liking” the A-Chair more (mean ± st. dev.: 7.27 ± 1.38), than the SS (5.00 ± 0.23); a statistically significant mean decrease of 2.27 (95% CI, −3.36, −1.186), (*t* = −4.66, *p* = 0.001) was obtained. For the rest of the comfort statements, no statistically significant differences were found, however, there was an overall positive trend across statements in favor of the A-Chair, with respect to the SS. These results highlight a slightly greater positive subjective perception of the A-Chair, with respect to the SS.

#### Helander and Zhang’s Discomfort Statements

Paired-sample *t*-tests were run on the nine statements for the 16 pianists to determine whether there were statistically significant mean differences in discomfort when using the A-Chair versus the SS (Figure 4B). Out of the nine statements, two were found to be statistically significant when comparing the two chairs. On one side, pianists agreed more to feeling more “cramped” when using the A-Chair (mean ± st. dev.: 2.19 ± 2.11), versus the SS (1.06 ± 0.25); a statistically significant mean decrease of 1.13 (95% CI, −2.15, −0.10), (*t* = −2.33, *p* = 0.034) was obtained. In contrast, pianists agreed more to feeling more “numbed” when using the SS (2.88 ± 1.86), than with the A-Chair (2.00 ± 1.37); a statistically significant mean decrease of 0.88 (95% CI, −0.014, 1.764), (*t* = 2.10, *p* = 0.05) was obtained.

When comfort and discomfort were assessed using the percentages of comfort (HZc) and discomfort (HZd), there was no association between these and the chairs (*t*-tests, HZc: *p* = 0.091; *t*-tests, HZd: *p* = 0.31).

#### Body Part Discomfort

General Linear Mixed Models (ANOVA with fixed factors: chair and side; random factor: assessed body parts) were used to explore the extent of pain experienced on the different body parts and the chairs. No statistical difference was found between the results associated to the A-chair and those associated to SS; however, the SS was found to cause slightly more pain (a difference of 0.5 points on a range of 10, on average) across body parts than with the A-Chair.

### Relationship Between Quantitative High Density Surface Electromyography and Qualitative Measurements (Musculoskeletal Questionnaires)

Spearman’s correlation was used to assess the relationship between the time change of the RMS_*ROA*_ and the GCR, HZc, and HZd. A correlation was found on the left side of the ESM between the change of the RMS_*ROA*_ on the A-Chair with respect to the SS and the GCR scores [*r* = 0.630; CI = (0.145, 0.871); *p* = 0.009], which highlights that at lower sEMG amplitude, the A-Chair is perceived to be more comfortable.

### Subcutaneous Adipose Tissue Thickness

The mean SAT on the right (R) and left (L) side was 8 mm and 9 mm, respectively. No significant differences were found between 12 measurements of the SAT on the R and L side of each subject at any of the three anatomical levels. The lack of significant differences between sides of the SAT thicknesses indicates that the RMS_*ROA*_ differences between R and L sEMG amplitudes and ROAs as well as between their percent changes are due to the neural drive, postural factors and chairs.

Finally, on the L side, the A-Chair was associated to a non-significant negative correlation, implying that the RMS_*ROA*_ decreased as the SAT thickness increased [*r* = −0.415, CI = (−0.765, 0.123), *p* = 0.124]; this non-significant correlation was also observed on the SS, with RMS_*ROA*_ decreasing as SAT thickness increased [*r* = −0.246, CI = (−0.674, 0.304), *p* = 0.376]. On the R side, the A-Chair was associated to a significant positive correlation between RMS_*ROA*_ and SAT thickness, with RMS_*ROA*_ increasing as SAT thickness increased [*r* = 0.579, CI = (0.095, 0.842), *p* = 0.024]; on the SS, a non-significant positive correlation was found with RMS values increasing as SAT thickness increased [*r* = 0.453, CI = (−0.078, 0.783), *p* = 0.090] (see section “General Considerations”).

## Discussion

### General Considerations

A novelty of this work is the use of a large electrode grid on each side of the spine and the definition of a ROA for each electrode grid to test the ESM of pianists. This eliminates the confounding factor due to the location of a single electrode pair, whose signal may be quite different depending on electrode location as evident from Figure 3. The need for HDsEMG technology stems also from previous work on violinists (Russo et al., 2019), and for other muscles, as indicated in the recent work of Vieira and Botter (2021). In previous reports, individual electrode pairs were placed on the back extensor muscles, including the Longissimus and Iliocostalis (Honarmand et al., 2018), with sEMG recordings for 10 min before and 10 min after 5 h of playing while the pianists were sitting on a standard piano stool or on a chair with back support. These authors also measured comfort using scales and concluded that “There were significantly lesser muscular activity, more ability to perform isometric back extension and better personal comfort while sitting on a chair with backrest.” Similarly, our exploratory work using a HDsEMG suggests that the use of a chair with lumbar support (A-Chair) ensuring a trunk-thigh angle of 115° ± 10° significantly reduces the RMS_*ROA*_ of sEMG detected over the ESM, in performing pianists, by (35.47 ± 21.46) % on the right side and (29.69 ± 23.01) % on the left side (Wilcoxon signed-rank test with *post hoc* Dunn’s *p* < 0.01).

Qualitative comfort changes indicate that the A-Chair provided greater comfort (when assessed using the GCR) than the SS. Additionally, the A-Chair was associated to more positive feelings with “likeliness” being significantly greater when compared to the SS. These qualitative results are found to be in line with the quantitative measurements of RMS and findings reported (Honarmand et al., 2018).

Two discomfort assessments were found to be of significance with the A-Chair causing greater feeling of “cramping” than the SS, and for the SS to cause a greater perception of “numbness” when compared to the A-Chair. No statistical difference was found by body part.

The lack of significant differences in the SAT values between sides of the ESM indicates that any RMS_*ROA*_ differences between sides cannot not be attributed to the SAT. Theoretically, if two measurements of sEMG RMS are performed using the SS and A-Chair one week apart, then SAT thickness, muscle and SAT-skin conductivities remained the same, the % variation of sEMG RMS should be independent of these three parameters. In this study, a significant correlation was observed between RMS_*ROA*_ values and SAT thickness on the right side of the ESM for both chairs, with RMS_*ROA*_ values increasing as SAT thickness increased. In contrast, on the left side, a correlation was found with RMS_*ROA*_ values decreasing as SAT thickness increased, but this was not statistically significant. The relation between RMS_*ROA*_ and SAT values requires further investigations that exceed the purpose of this work.

Individual comfort/discomfort, as well as sEMG RMS_*ROA*_ amplitude and PRMD, are not only related (or caused by) muscular activity: they can also be due to the mechanical conditions of the intervertebral disks and the curvature of the spine, clinically associated to back pain (Laird et al., 2014; Chun et al., 2017). This issue is not addressed in this work because no subject had back pain and clinical considerations were not the focus of this work. The literature concerning ergonomics and occupational medicine usually considers sEMG as an index of exposure, quantified through the Exposure Variation Analysis (EVA), whose values should be reduced to reduce exposure and risk (Hägg et al., 2000; Reynolds et al., 2014). However, this technique still finds limited use among clinicians and deserves further exploration.

The burst-like patterns observed on the HDsEMG of the ESM were not related to either side or chair type (visual observations). Similar patterns have been studied in sitting violinists (Khorrami Chokami et al., 2021) and should be further investigated.

### Comparison With Previous Work

Our results are in agreement with those obtained in previous work on sitting violinists (Russo et al., 2019) and show that the use of a chair with lumbar support (such as A-Chair) has a significant impact on the RMS_*ROA*_ value measured from the ESM on both violinists and pianists. When using the A-chair rather than the standard orchestra chair, nine out of nine violinists showed a significantly lower RMS_*ROA*_ on both the R and L sides (Wilcoxon signed rank test). When using the A-chair rather than the piano stool, 14 out of 16 pianists showed a lower RMS_*ROA*_ on the L side and R side (all statistically significant, Wilcoxon signed rank test). The probability of this happening by chance, according to the binomial distribution, is less than 0.004 in each case. Other qualitative findings are similar in the two studies.

Burst-like ESM activation is more evident in the violin players (eight out of nine subjects) (Russo et al., 2019) than in the piano players (seven out 16 subjects). This issue deserves further investigation.

## Conclusion and Limitations of the Study

### Conclusion

This is the first work addressing the ESM of pianists with large electrode grids providing overall topographical maps. As shown for other muscles (Vieira and Botter, 2021), the HDsEMG approach is expected to identify the effect of two chairs on the ESM of pianists better than a single electrode pair that provides only a local sample. A total of eleven previous publications deal with HDsEMG analysis of back muscles, out of which three are from our own group, with others mostly addressing lower back pain, myofascial trigger points, lifting exercises, or other physiological investigations. Other authors have further investigated the ESM of pianists using only one or a few electrode pairs.

The relevance of HDsEMG is confirmed by Figure 3 which shows that the map of RMS amplitude over the ESM is not uniform and the signal intensity is stronger near the spine and not homogeneously distributed, confirming previous findings (De Nooij et al., 2009) and supporting the observations of Vieira and Botter (2021) about the fact that using single electrode pairs may lead to different conclusions depending on the location of the electrode pair on the muscle. The spatial average of the sEMG RMS over a ROA provides an indication of the ESM activity more reliable than that provided by a single electrode pair.

There was a significant difference between the RMS_*ROA*_ of the right and left side of the ESM. This difference may be due to hand dominance and deserves further investigation.

It is also concluded, from the relationship between quantitative and qualitative measures, that using a chair with lumbar support and a trunk-thigh angle of 115° ± 10° provides greater comfort, less exertion of the ESM, and less discomfort than the standard piano stool. This is in agreement with the findings of a previous work on sitting violinists (Russo et al., 2019).

Finally, a decreasing trend of mean RMS was observed over time, but no significant change of the ROA’s centroid was observed possibly because the inter-subject variability was considerable.

### Limitations of the Study

#### Normalization

Actual sEMG RMS values (expressed in μV) are used in this study to estimate differences between chairs and sides, expressed as percent changes. They have not been normalized with respect to a reference value (e.g., the value associated to the maximal voluntary contraction of the ESM). On the other hand, normalization would not change the relative variations of RMS_*ROA*_ due to chairs reported in Table 2.

#### Noise Baseline

It is good practice to estimate the background noise level in every subject, in relaxed conditions, before testing. Because of the limited time availability of the subjects this was replaced by a separate estimate of background noise measured on the ESM on a test group of four subjects lying prone on a bed, as described in section “Electrode Placement, HDsEMG Recording and Processing.” Noise measurements were repeated 12 times (for 20 s each time) every 5 min. Twice the mean RMS_*ROA*_ value was taken as the noise level and RMS_*ROA*_ was considered reliable if >5 μV_*RMS*_.

#### Music Played

The same piece of music was played by all subjects during each 20 s test. The relationship between sEMG and the music type and speed were not investigated and no metronome was used.

#### Sample Size and Homogeneity of the Sample

The number of subjects was limited by availability but significant differences in sEMG were found in 14 subjects in favor of the A-chair (see section “Results”). The sample of investigated subjects was not homogenous and was not large enough to allow investigation of the effects of sex, age, and experience. However, the fact that the chair effect is so clearly evident, suggests that it is present across sexes, ages and expertise/years of practice. Our results justify larger studies, focused on these confounding factors. In this work, the effect of the chair stands out of these factors.

#### No Randomization

For organizational reasons, in all cases, the test on the SS chair was done first and the test on the A-chair was done one week later. It was not possible to blind the pianists to the two type of chairs. However, in crossover studies (such as this one), one new treatment (A-Chair) is tested against a standard treatment (SS) that has been used for a long time. With this type of studies, every participant serves as its own control. The order of testing cannot be randomized. If we take into account that each subject has played the piano a minimum of 7 h and up to 34 h a week using the SS, then the session on A-Chair could never be done before using the SS, which has been used for at least 5 years prior to the day these measurements took place. For this reason, the only factors that we could possibly change by changing the order of testing would have been any psychological effects (e.g., application of electrodes, familiarization with experimenters). It is unlikely that these factors influenced the sEMG. Finally, it has been demonstrated in other studies that one week between tests provide a minimal memory effect, yet short enough to avoid changes in muscle strength (Spijkerman et al., 1991).

#### Spectral Parameters

Surface EMG spectral features (mean or median frequency of the sEMG power spectrum) were investigated. In our work the signal/noise ratio appeared to be too small for a reliable estimate of spectral variables.

## Data Availability Statement

The data will be available upon resonable request.

## Ethics Statement

The studies involving human participants were reviewed and approved by the Italian National Health Service. Written informed consent to participate in this study was provided by the participants’ legal guardian/next of kin. Written informed consent was obtained from the individual(s) for the publication of any potentially identifiable images or data included in this article.

## Author Contributions

AR, FS, SD’E, and AA-G collected the data. AR and AA-G performed the analyses. AA-G prepared the manuscript. RM supervised the work. All authors read and provided feedback on the manuscript.

## Conflict of Interest

FS was employed by company Montecatone Rehabilitation Institute S.p.A. The remaining authors declare that the research was conducted in the absence of any commercial or financial relationships that could be construed as a potential conflict of interest.

## Publisher’s Note

All claims expressed in this article are solely those of the authors and do not necessarily represent those of their affiliated organizations, or those of the publisher, the editors and the reviewers. Any product that may be evaluated in this article, or claim that may be made by its manufacturer, is not guaranteed or endorsed by the publisher.
